# Supplementary material for: Content-rich biological network constructed by mining PubMed abstracts
Source: BMC Bioinformatics. 2004 Oct 8;5:147. doi: 10.1186/1471-2105-5-147 (PMC528731; doi:10.1186/1471-2105-5-147)
Supplement: Additional File 5 — The original Chilibot query results of the term "long-term potentiation (LTP)" and 22 other terms, limiting the latest references analyzed to the years 1990, 1995, 2000, and 2004. [file 1471-2105-5-147-S5.bz2 › chilibotAdditionalFile5/ltp2000/html/LTP_TRKA.html]

 


 **LTP** and **TRKA** 
  
Found 3 abstracts in PubMed,  **3 abstracts were retrieved and analyzed**.  


---

 Search Google  |
 PDF files only 
|  EDU domain only 

---

**Interactive relationship** (e.g. stimulation, inhibition, etc)

- At later postnatal ages, blockade of either endogenous NGF by immunoadhesin  **TrkA**  IgG or  **TrkA**  receptors by monoclonal antibody rescues  **LTP** .  Ref: 10707981 Neuron, 2000
- Blocking the NGF  **TrkA**  interaction rescues the developmental loss of  **LTP**  in the rat visual cortex role of the cholinergic system.  Ref: 10707981 Neuron, 2000

**Parallel relationship** (e.g. studied together, co-existance, homology, etc.)

- These findings are consistent with the hypothesis that NGF, by interacting with  **trkA** , triggers a sequence of tyrosine kinase dependent phosphorylation steps that modulate glutamate release and calcium influx and impact on expression of  **LTP**  in dentate gyrus.  Ref: 10560922 Hippocampus, 1999
- Analysis of phosphorylation of the two tyrosine kinase substrates  **trkA**  and ERK in synaptosomes prepared from untetanized and tetanized dentate gyrus revealed that  **LTP**  was associated with increased phosphorylation of both proteins.  Ref: 10560922 Hippocampus, 1999
